# Oral health-related quality of life of patients after solid organ transplantation is not affected by oral conditions: results of a multicentre cross-sectional study

**DOI:** 10.4317/medoral.24277

**Published:** 2020-12-19

**Authors:** Gerhard Schmalz, Jens Garbade, Urte Sommerwerck, Otto Kollmar, Dirk Ziebolz

**Affiliations:** 1 Department of Cariology, Endodontology and Periodontology, University of Leipzig, Germany; 2 University Department of Cardiac Surgery, Heart Center Leipzig, Germany; 3 Department of Pneumology, Allergology, Sleep and Respiratory Medicine, Hospital Augustinerinnen - Severinsklösterchen, Cologne, Germany; 4 Universitäres Bauchzentrum Basel, Universitätsspital Basel, Switzerland

## Abstract

**Background:**

This multicentre cross-sectional study aimed in examination of oral health-related quality of life (OHRQoL) of patients after solid organ transplantation (SOT).

**Material and Methods:**

Patients after SOT (liver, lung and heart) at one out of three German centers (Goettingen, Essen, Leipzig) were included. For comparison, a healthy control (HC) was recruited. OHRQoL was assessed by German short form of oral health impact profile (OHIP G14). Oral examination comprised: decayed-, missing- and filled-teeth index (DMF-T), remaining teeth and periodontitis severity.

**Results:**

In total, 196 patients after SOT and 130 HC with comparable age, gender and smoking habits were included (*p*>0.05). DMF-T and number of remaining teeth was worse in SOT group (*p*<0.01). OHIP G14 sum score was significantly higher in SOT (3.49 ± 5.73 vs. 1.33 ± 2.63, *p*<0.01). In contrast to HC, in SOT no associations between OHIP G14 and oral health parameters were found (*p*>0.05). Number of remaining teeth was not an independent predictor of OHIP G14 sum score in SOT (β -0.082, CI95 -0.156 - 0.045, *p*=0.28).

**Conclusions:**

OHRQoL of SOT recipients is not affected by their oral condition, leading to the assumption that the individual perception of patients physical oral health is not in line with the clinical situation.

** Key words:**Oral health, oral health-related quality of life, solid organ transplantation.

## Introduction

Due to an increased risk of systemic infectious complications related to the lifelong immunosuppressive therapy, dental care of patients after solid organ transplantation (SOT) is of high clinical relevance (1). However, patients after SOT often suffer from a high prevalence of dental and periodontal diseases as well as oral mucosal lesions and reduced oral behaviour (2-4). While it was reported that SOT recipients show a high dental and periodontal treatment need, irrespective of their time since transplantation or their form of immunosuppression, sufficient pre- and post-transplant dental care concepts are missing (2,5,6). Accordingly, a lack in dental care of SOT recipients appears evident. An observational study with 12 months follow-up after dentist allocation of patients after heart transplantation revealed more than 70% of patients to show periodontal treatment need, although they had visited a dentist, underlining the lack of appropriate dental care of these patients (7).

An important parameter related to oral conditions is the oral health-related quality of life, which reflects the patient´s perception of his oral status (8). It has been repeatedly demonstrated that patients after SOT, including liver, lung, kidney and heart transplantation, show a nearly unaffected OHRQoL, irrespective of their high dental and periodontal treatment need or oral disease burden, respectively (9-13). Therefore, it might be assumed that patients after SOT show an altered perception of their oral conditions, which is not in line with the clinical situation. In generally healthy individuals, periodontitis and especially tooth loss is related to a reduced OHRQoL (14,15). Accordingly, the available results of patients after SOT appear contradictory to this. However, the perception of their insufficient oral health situation as not restrictive for OHRQoL might be a possible explanation for patients´ reduced dental behaviour, making this issue relevant for the dental care of SOT recipients. Up until now, it is unclear whether this phenomenon is generally derivable for patients after SOT.

Therefore, the aim of this current cross-sectional study was to assess OHRQoL of a large cohort of SOT recipients, including liver, lung and heart transplantation in comparison to a healthy control group. Thereby it was aimed to detect, whether the OHRQoL of patients after SOT is associated to oral health and general parameters, especially with regard to the number of remaining teeth. It was hypothesised that OHRQoL of SOT recipients would be at most slightly reduced compared to the HC and not associated to oral conditions in the SOT group.

## Material and Methods

- Study design

This current multicentric cross-sectional study included patients with different solid organ transplants, including liver (LTx), lung (LuTx) and heart transplantation (HTx). The included patients were part of different clinical investigations, which were all reviewed and approved by the respective local ethics committees of the University Medical Center Goettingen (LTx: No: 29/1/14), University Hospital Essen (LuTx: No: 13-5689-BO) and University of Leipzig (HTx: No: 414/16-ek). The study has been performed in accordance with the ethical standards laid down in an appropriate version of the 2000 Declaration of Helsinki as well as the Declaration of Istanbul 2008. Furthermore, the “Strengthening the Reporting of Observational Studies in Epidemiology (STROBE)” guidelines for reporting observational studies were followed (16). All participants were informed verbally and in writing about the planned studies and gave their written informed consent for participation. Furthermore, guidelines for ethical approvals for human subjects were followed in accordance with the Declaration of Helsinki. Some of the included patients were also still part of previous examinations by this working group (2,9-12).

- Patients

No previous power calculation was applied; all available patients, who met the in- an exclusion criteria should be included in the study. Participants in SOT group were composed from three different German transplant centers. Patients with LTx were recruited within a regular follow-up appointment at the transplant outpatient clinic of the University Medical Center Goettingen. LuTx patients were included during their routine outpatient visits to the lung transplant unit of the Ruhrlandklinik, Essen. HTx patients who attended the University Department for Cardiac Surgery, Leipzig Heart Centre, Leipzig, were recruited for the study during their routine follow-up appointment. Generally, only patients with an age of at least 18 years were included. As general exclusion criteria of the respective studies, impossibility to undergo clinical examination due to a worse general health status, autoimmune diseases (e.g., rheumatoid arthritis), infectious diseases (e.g., hepatitis A, B, C, tuberculosis, or HIV) and pregnancy were determined. The patient-related general data, including age, gender, smoking (smoker or non-smoker), time since SOT and presence of diabetes mellitus were extracted from the patient records. For comparison, a healthy control group (HC) with patients without SOT from the Department of Cariology, Endodontology and Periodontology, University of Leipzig, Germany was included. Thereby, generally healthy individuals with comparable age, gender and smoking habits like the SOT patients were selected (matching), as far as possible.

- Oral health examination

All participants underwent a standardized oral examination by skilled and experienced dentists at the three transplant centers (SOT group) or the Department of Cariology, Endodontology and Periodontology, University of Leipzig (HC group). To assess the dental condition, the decayed-, missing- and filled-teeth index (DMF-T) was applied according to WHO (17). Therefore, teeth with a cavitation of the dental hard tissues were added as D-T, filled and crowned teeth were assigned to F-T component and missing teeth were added as M-T. Furthermore, the number of remaining teeth was recorded. To draw conclusions on the severity of periodontal disease burden, patients underwent a periodontal examination. Thereby, periodontal probing depth and clinical attachment level were recorded at six measurement points per tooth by using a millimetre scaled periodontal probe (PCP 15, Hu-Friedy, Chicago, IL, USA). Based on this measurement, periodontitis was classified into no/mild, moderate or severe periodontitis (18).

- Oral health-related quality of life

To assess the OHRQoL, the German short form of the Oral Health Impact Profile (OHIP G14) was applied (19,20). This standardized and validated questionnaire includes questions regarding 14 functional and psychosocial impacts that participants perceived in the previous month related to problems with their teeth, mouth or dentures. On a five-point scale between 0-4: very often = “4”, fairly often = “3”, occasionally = “2”, hardly ever = “1”, and never = “0”, questions can be answered by the patient, resulting in a higher score indicating a worse OHRQoL. Alongside with the total sum score of the OHIP G14, four different dimensions, “oral function”, “psychosocial impact”, “oral pain” and “orofacial appearance”, were considered (21). Out of these dimensions, only oral function and psychosocial impact were chosen to analyse potential associations between oral health and general parameters with OHRQoL.

- Statistical analysis

All statistical analyses were performed with SPSS for Windows, version 24.0 (SPSS Inc., USA). The Kolmogowov-Smirnov test did not confirm any metric variable to be normal distributed (*p*<0.05); therefore, non-parametric tests for non-normal distributed samples were applied. Two independent, non-normal distributed samples were analysed by Mann-Whitney-U test. More than two independent, non-normal distributed parameters were compared with Kruskal-Wallis test. Categorical data were analysed by chi-square or fisher test, respectively. A regression analysis was applied to detect, whether remaining teeth would be an independent predictor of worse OHIP G14 sum score.

## Results

- Patients

In SOT group, a total of 196 patients (LTx: 63, LuTx: 66, HTx: 67) with an average time of 5.83 ± 4.38 years since SOT were included. The HC consisted of 130 individuals. The age (SOT: 55.76 ± 11.45 vs. HC: 56.08 ± 10.75, *p*=0.88), gender (61.2% male vs. 60.8% male, *p*=0.90) and smoking habits (28.1% smoker vs. 24.6% smoker, *p*=0.52) were comparable between groups. About 40% of SOT patients had a diabetes mellitus, while none of the HC individuals had diabetes (*p*<0.01).

- Oral health examination

**Figure 1:**
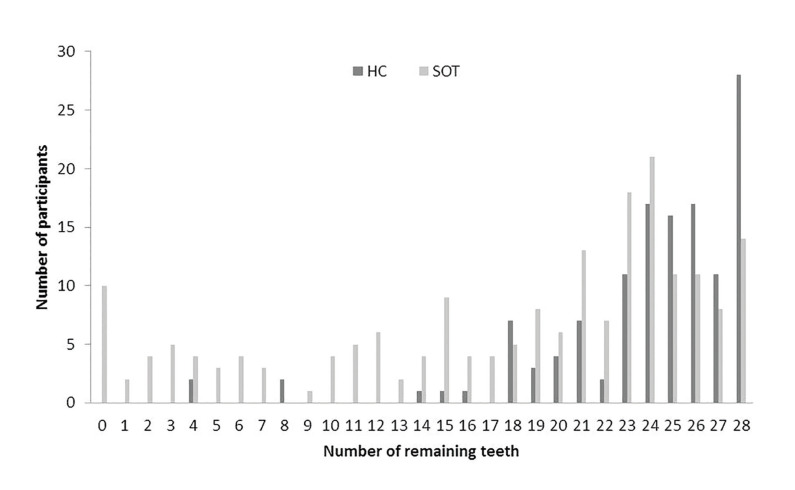
Distribution of remaining teeth in the two groups after solid organ transplantation (SOT) and healthy control (HC).

The DMF-T was statistically significant higher in the SOT compared to HC group (19.98 ± 6.93 vs. 16.76 ± 6.67, *p*<0.01). The prevalence of carious lesions (D-T) was also higher in SOT group (0.77 ± 1.64 vs. 0.22 ± 0.99, *p*<0.01). With 17.87 ± 8.46 remaining teeth, SOT group had significantly less remaining teeth than HC (23.90 ± 4.54, *p*<0.01). Thereby, the majority of HC had more than 20 remaining teeth, while in SOT a large amount had less than 20 remaining teeth (Fig. 1). The periodontal disease severity was comparable between groups (*p*=0.60, Table 1).

**Table 1:**
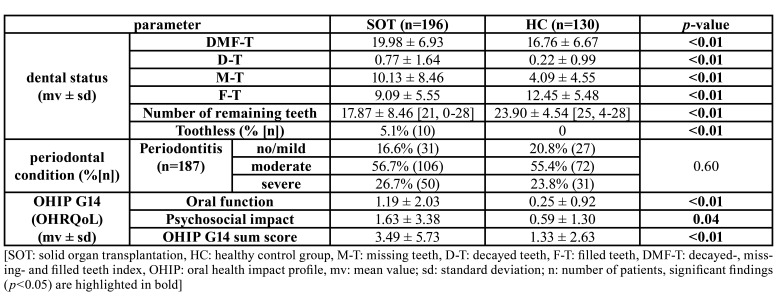
Oral health and OHIP sum scores between groups.

- Oral health-related quality of life

The OHIP G14 sum score was clinically relevant and statistically significant higher in SOT compared to HC group (3.49 ± 5.73 vs. 1.33 ± 2.63, *p*<0.01). Also the scores of the dimensions oral function (1.19 ± 2.03 vs. 0.25 ± 0.92, *p*<0.01) and psychosocial impact (1.63 ± 3.38 vs. 0.59 ± 1.30, *p*=0.04) were significantly higher in SOT group (Table 1). Out of the different questions, “trouble pronouncing” (*p*=0.02), “taste worsened” (*p*<0.01), “life less satisfying” (*p*=0.02), “interrupting meals” (*p*=0.01), “uncomforTable to eat” (*p*<0.01), “short tempered” (*p*=0.01), “difficulty performing jobs” (*p*=0.01), “embarrassed” (*p*=0.02) and “sense of uncertainty” (*p*=0.02) were significantly worse in SOT patients (Table 2).

**Table 2:**
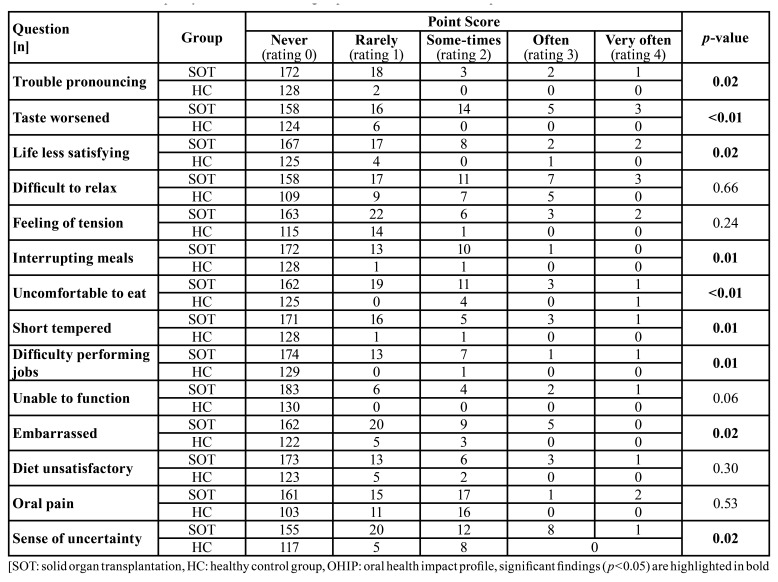
Oral health-related quality of life between both groups assessed with OHIP G14 questionnaire.

- OHRQoL and oral health as well as general parameters

In the SOT group, neither oral health nor general parameters were found to be associated to OHIP G14 sum score as well as dimensions oral function and psychosocial impact (pi>0.05; Table 3). Moreover, the number of remaining teeth was not an independent predictor of OHIP G14 sum score in SOT group (β -0.082, CI95 -0.156 - 0.045, *p*=0.28). In the HC group, DMF-T (*p*<0.01), remaining teeth (*p*<0.01), prevalence of severe periodontitis (*p*=0.02) and age (*p*=0.01) were associated to OHIP G14 sum score, which was also found for the dimension psychosocial impact (Table 4). The dimension oral function was associated to DMF-T (*p*<0.01), remaining teeth (*p*<0.01) and age (*p*<0.01; Table 4). In HC, the number of remaining teeth was an independent predictor of OHIP G14 sum score (β -0.322, CI95 -0.458 - -0.083, *p*<0.01).

**Table 3:**
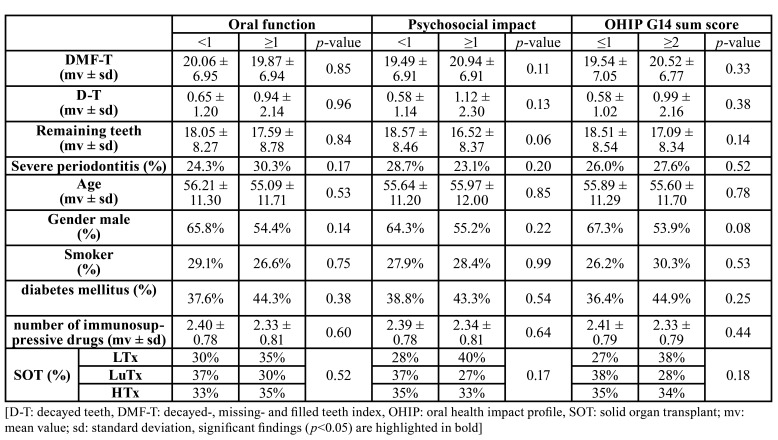
Associations between oral and general parameters with OHIP G14 sum score, as well as dimensions oral function and psychosocial impact in SOT group.

**Table 4:**
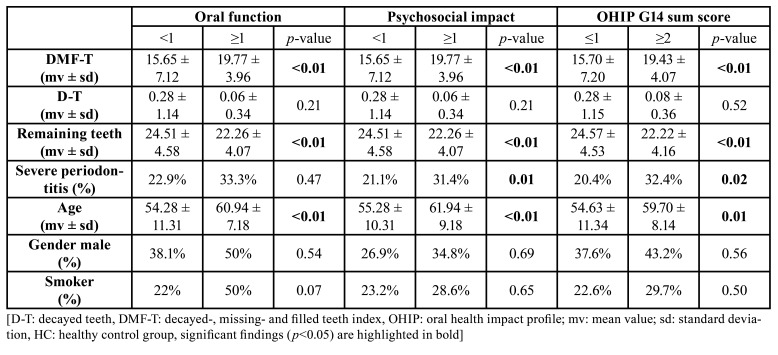
Associations between oral and general parameters with OHIP G14 sum score, as well as dimensions oral function and psychosocial impact in HC group.

## Discussion

The patients after SOT in the current study were found to show worse dental health and a slightly worse OHRQoL than the HC. While SOT recipients showed an OHRQoL, which was not associated to oral conditions, participants in HC had associations between oral health and OHRQoL. Thereby, the number of remaining teeth was an independent predictor of OHRQoL in HC, but not in SOT group.

It has been repeatedly reported, that oral health situation of patients after SOT would be worse compared to generally healthy individuals or the generally healthy population, respectively (2-4). This is in line with the current study´s findings. However, the high periodontal burden of SOT recipients, as shown in the literature (4,7,22), appears not to be significantly higher than in healthy controls. In this respect, the high prevalence of periodontal treatment need in German general population, reflected in the fifth German oral health study should be considered (23). Accordingly, periodontal disease burden appears comparably high between generally healthy and SOT individuals. Therefore, the demand of an early pre-transplant dental rehabilitation and a post-transplant dental maintenance, as demanded in literature for several decades appears still contemporary and necessary (1,2,24). The lower number of remaining teeth in SOT compared to HC might indicate a surgical dental clearance before transplantation, whereby the major focus seems on tooth extraction instead of restoration. This partly rigorous procedure can be discussed controversially, especially due to limited evidence (5).

To expand the horizon of physical oral health parameters, patient-reported outcome measures, like the OHRQoL are a mandatory part of evidence based dentistry and research (8). Therefore, this clinical study applied the OHIP G14 as a validated and standardized questionnaire, which is suiTable for research questions (8). The comparison of OHIP G14 sum scores between SOT and control group shows a statistically significant and clinically relevant difference between the two groups, if the principle of minimal important difference is followed (8). To reflect the overall impairment of OHRQoL in the SOT group, the findings must be interpreted with regard to reference values for German general population. John *et al*. 2004 found reference values depending on dentition of a sum score between 0 (fully dentate) and 6 (edentulous, wearing full dentures) points (25). The current study included patients irrespective of their number of teeth and found an average sum score of 3.49 points, which is completely within the reference range. Accordingly, the OHRQoL of the SOT cohort in the current study can be interpreted as unaffected. This is in line with previous studies on OHRQoL of patients after kidney Tx (9), LuTx (10) and LTx (11). In contrast, three other studies found a slightly reduced OHRQoL of patients after HTx or kidney Tx, respectively (12,13,26). Altogether, a nearly unaffected OHRQoL of patients after SOT can be assumed based on the literature and the current study´s results. Furthermore, the current study analysed two dimensions of OHIP G14 including oral function and psychosocial impact. The analysis of these dimensions showed a significant difference between SOT and HC; however, the difference was only minor and thus a clinically relevant impairment of oral function or psychosocial impact dimension appears not to be present in SOT recipients.

Regularly, the OHRQoL is affected by dental and periodontal health, whereby especially the number of missing/remaining teeth is an important influential factor on the perception of the oral conditions (14,15). Accordingly, it is not surprising that the healthy control group was found to show associations between dental health and OHRQoL. Moreover, number of remaining teeth was an independent predictor of OHRQoL. This is supported by the recent literature, where especially the number of remaining functional pairs of antagonists is a relevant factor for OHRQoL (15,27). While the healthy control group showed these expecTable results, for SOT recipients no associations to dental or periodontal conditions were confirmed. However, this is quite in line with the available literature, where only one study found physical oral health to be related to OHRQoL in patients after kindey Tx (28), while five other studies did not find an association between OHRQoL and physical oral health (9-13). The current study did not confirm any hints that OHRQoL of SOT patients would be affected by general or SOT related parameters; neither age, gender or smoking habits, nor diabetes mellitus, transplanted organ or the number of immunosuppressive drugs were associated to OHRQoL. Considering the high prevalence of dental and periodontal diseases in SOT recipients, it appears exceptional, that these oral conditions seem not to affect the OHRQoL. The presence of remaining teeth was not found to be a predictor for the OHRQoL of SOT recipients, although this is one of the strongest influential factors on OHRQoL in regular case.

Accordingly, it might be hypothesized that these patients are affected in their individual perception of the clinical oral conditions. This might be explained by a phenomenon, which is similar to a “response-shift”, what mediates an adaption or accommodation of a chronic disease process (29). Due to the severe disease causing necessity of SOT and the lifelong immunosuppression and medical care, patients with SOT might accommodate a status of being “chronically ill”, resulting in a shift of health concerns, including diseases of the oral cavity. Thereby, patients would not perceive their insufficient oral condition as a problem, what might result in reduced dental behaviour and thus into a vicious circle of reduced oral behaviour and worse dental/periodontal health. Although this remains just speculative, this would be an important approach for dental care and necessary to improve oral health situation of SOT recipients.

Strengths and limitations: up until now, this is largest clinical examination of SOT recipients regarding their OHRQoL and the first study, in which different groups of transplanted organs are considered. Although no sample size calculation was performed, the inclusion of 196 patients appears a reasonable cohort, which allows robust conclusions. The inclusion of a healthy control for comparison, application of OHIP G14 questionnaire as a valid instrument and the consideration of physical oral health findings are further strengths. The study is limited by their cross-sectional design, making causative conclusions impossible. Moreover, the oral examination limits the ability to draw more detailed conclusions; thereby, the number of remaining molars or functional occlusal pairs might have been meaningful. Furthermore, the SOT cohort is very heterogeneous with regard to the respective organ, underlying disease, co-morbidities and medication, what limits the generalizability of the findings.

## Conclusions

The OHRQoL of SOT recipients lies within the reference for general healthy population and was just slightly lower than in the included healthy control group. Thereby the OHRQoL of SOT recipients is not affected by their oral condition, what leads to the assumption that the individual perception of patients physical oral health is not in line with the clinical situation. Therefore, the sensibilisation for the oral situation and its relevance for general health appears an important issue for improving dental care of patients after SOT.
